# Supplementary material for: Hibernating bear serum hinders osteoclastogenesis in-vitro
Source: PLoS One. 2020 Aug 27;15(8):e0238132. doi: 10.1371/journal.pone.0238132 (PMC7451522; doi:10.1371/journal.pone.0238132)
Supplement: Attachment — Submitted filename: 2020 bear serum review.docx [file pone.0238132.s001.docx]

This is an interesting study that is consistent with osteoclastogenesis in peripheral blood mononuclear cells being influenced by serum factors in hibernating and active bears. This builds on some previous research with chicken osteoclastogenesis being affected by bear serum and the observation that bears do not suffer significant bone loss during hibernation. There are some troubling experimental design flaws in the paper that should be addressed before publication.

Introduction

Line 72: ground squirrels do not eat during hibernation and rarely urinate or defecate.

Line 80: it would be good to compare and contrast bears to what is known about bone loss in other hibernators such as ground squirrels. For example: McGee-Lawrence ME, Stoll DM, Mantila ER, Fahrner BK, Carey HV, Donahue SW. Thirteen-lined ground squirrels (Ictidomys tridecemlineatus) show microstructural bone loss during hibernation but preserve bone macrostructural geometry and strength. *J Exp Biol* 214: 1240–1247, 2011.

Line 82: change human to humans

Methods

Line 123: add “have” in front of “access” in both occurrences

Line 129: was blood collection done similarly for both hibernating and alert animals? Some anesthetics don’t work as well on hibernating animals due to decreased circulation and metabolism

Line 131: if blood was collected in EDTA this would produce plasma. Was clotting initiated to form serum? Or was plasma actually used in the experiments.

Line 174: Change “Next day of” to “The day after”

For the experimental design (table 1 and lines 188-194)

I have a few concerns with the experimental design.

1. While an n=3 for PBMCs is fairly low an n=1 for ADSC is unacceptably low. It also appears that individual PBMC cell preps were incubated with serum from individual animals. Were any experiments performed where a single cell prep was incubated with each of the different serum samples to ensure there were not differences between serum samples. Or a pooled serum sample could be used in common with all three cell preps. As the experiment is designed it is difficult to tell if any variation is due to the cell prep or the serum.
2. The difference in growth between active (ABSM) and hibernating (HBSM) sera is interesting and suggests that ABSM contains an activator or HBSM contains an inhibitor of PBMC differentiation. A classical experimental method to test this is to either heat inactivate each serum or do a mixing experiment. For example if you heat inactivate ABSM and growth no longer occurs it could contain an activator, while if you heat inactivate HBSM and growth does occur then it contained an inhibitor. Similarly, if HBSM contains an inhibitor, adding it to ABSM could decrease growth. There are multiple ways these experiments may not work (heat stable factors, etc.) but they are fairly simple and broad experiments that would allow the authors to draw a more substantial conclusion about their results.

Results: The images are not of great quality, but if they meet the standards of the journal they should be sufficient. My major concern with the results are addressed above regarding the sample size, especially for ADSC, and the mixing and heat inactivation controls.

Discussion: A quick summary of the use of PBMCs in in vitro osteoclastogenesis should be added to the introduction. My first thought when reading the introduction is whether this model was physiologically relevant, i.e. how many osteoclasts arise from PBMCs vs. bone marrow. If this is the standard of the field it would be good to mention this up front.

The mixing and heat inactivation experiments are even more important to perform given the mention on line 296 that hibernating serum inhibits chicken OCs formation.

The authors come very close to concluding that HBSM might contain an OC inhibitor in lines 304-313. The experiments described above would allow them to test this hypothesis and make this conclusion directly.

The rest of the discussion speculates on possible factors that may be lacking in hibernating serum. I think the authors would be advised to first determine if an activator is lacking or an inhibitor is present before testing individual potential lead compounds.
