# Supplementary material for: Hibernating bear serum hinders osteoclastogenesis in-vitro
Source: PLoS One. 2020 Aug 27;15(8):e0238132. doi: 10.1371/journal.pone.0238132 (PMC7451522; doi:10.1371/journal.pone.0238132)
Supplement: Attachment — Submitted filename: bear osteogenesis.docx [file pone.0238132.s003.docx]

The authors addressed my concerns and attempted heat inactivation and mixing experiments. 65C is probably too high a temperature for serum inactivation, for tissue culture heat inactivation at 55C for 30 minutes is traditional. For mixing experiments it is important to keep the final concentration of serum the same, so 5% of each to give a total of 10%. This is how assays for clotting factor deficiencies or inhibitors are done. Serum samples could also be pooled, similar to a pooled normal plasma, to reduce the number of experiments that need to be done. For future studies this approach could be used to determine if the authors are looking for an inhibitor or an activator, the first step in determining a mechanism of action.
